# *Libidibia ferrea* Fruit Crude Extract and Fractions Show Anti-Inflammatory, Antioxidant, and Antinociceptive Effect* In Vivo* and Increase Cell Viability* In Vitro*

**DOI:** 10.1155/2019/6064805

**Published:** 2019-02-21

**Authors:** Tamires Rocha Falcão, Aurigena Antunes de Araújo, Luiz Alberto Lira Soares, Iuri Brilhante de Farias, Wliana Alves Viturino da Silva, Magda Rhayanny Assunção Ferreira, Raimundo Fernandes de Araújo Jr, Juliana Silva de Medeiros, Maria Luiza Diniz de Sousa Lopes, Gerlane Coelho Bernardo Guerra

**Affiliations:** ^1^Postgraduate Program in Pharmaceutical Science, Department of Pharmacy, UFRN, Natal, RN, Brazil; ^2^Postgraduate Program in Public Health, Postgraduate Program in Pharmaceutical Science, Department of Biophysics and Pharmacology, UFRN, Natal, RN, Brazil; ^3^Laboratory of Pharmacognosy, Postgraduate Program in Therapeutic Innovation, Department of Pharmaceutical Sciences, UFPE, Recife, PE, Brazil; ^4^Department of Pharmaceutical Sciences, UFRN, Natal, RN, Brazil; ^5^Postgraduate Program in Functional and Structural Biology, Postgraduate Program in Health Science, Department of Morphology, UFRN, Natal, RN, Brazil; ^6^Postgraduate Program in Functional and Structural Biology, Department of Morphology, UFRN, Natal, RN, Brazil; ^7^Postdoctoral Fellow in the Postgraduate Program in Public Health, Department of Dentistry, UFRN, Natal, RN, Brazil; ^8^Post Graduation in Biological Science, Post Graduation Program in Pharmaceutical Science, Department of Biophysics and Pharmacology, UFRN, Natal, RN, Brazil

## Abstract

**Background:**

* Libidibia ferrea* (*L. ferrea) *is found throughout the northeastern region of Brazil, where it has been used in folk medicine with beneficial effects on many inflammatory disorders.

**Purpose:**

This study investigated the phytochemical composition of the crude extract and fractions of* L. ferrea* fruit and evaluated its anti-inflammatory and antinociceptive activities* in vivo* and effect on cell viability* in vitro*.

**Methods:**

Characterization of polyphenols present in crude extract (CE), hydroalcoholic fractions of 20-80% ethanol (CE20, CE40, CE60, and CE80), aqueous fraction (AqF), and ethyl acetate (EAF) fractions of* L. ferrea* fruit was performed by chromatographic analysis. Anti-inflammatory activity was evaluated by using a carrageenan-induced peritonitis model submitted to a leukocyte migration assay and myeloperoxidase activity (MPO) analysis. Total glutathione and malondialdehyde (MDA) levels were assessed to evaluate the oxidative stress level. Antinociceptive activity was evaluated by acetic acid-induced abdominal writhing and hot plate test.* In vitro *cell viability was determined by using MTT assay in a mouse embryonic fibroblast cell line (3T3 cells).

**Results:**

Chromatography revealed the presence of ellagic acid content in EAF (3.06), CE (2.96), and CE40 (2.89). Gallic acid was found in EAF (12.03), CE 20 (4.43), and CE (3.99).* L. ferrea* crude extract and all fractions significantly reduced leukocyte migration and MPO activity (p<0.001).* L. ferrea* antioxidant effect was observed through high levels of total glutathione and reduction of MDA levels (p<0.001). Acetic acid-induced nociception was significantly inhibited after administration of* L. ferrea* crude extract and all fractions (p<0.001). Crude extract and all fractions significantly increased the viability of the 3T3 cell line (p<0.05).

**Conclusions:**

The appropriate extraction procedure preserves the chemical components of* L. ferrea* fruit, such as gallic acid and ellargic acid. Crude extract and fractions of* L. ferrea* fruit exhibited anti-inflammatory, antioxidant, antinociceptive activities* in vivo *and enhanced cell viability* in vitro*.

## 1. Introduction

*Fabaceae* (*Leguminosae*) is one of the most economically important botanical families. There are many useful species and numerous amounts of them have been cultivated since ancient times, mainly due to their food and medicinal potential, although there are several other utilities.* Libidibia ferrea (L. ferrea)*, popularly known as jucá or pau-ferro, is a species of the* Leguminosae* family with multiple medicinal uses [1]. Studies performed with species of the* Leguminosae* family have demonstrated antihelmintic, antimalaria, anti-inflammatory, and analgesic activity [2–5].

*L. ferrea* occurs throughout the northeastern region of Brazil [6].* L. ferrea* is popularly used to treat diabetes and rheumatism and presents hepatoprotective, antifertility, analgesic, anti-inflammatory, and cardiovascular action [7]. There are several therapeutic properties described in folk medicine for* L. ferrea* fruit [8]. Bacchi et al. [9, 10] described the effect of its crude aqueous extract against gastric ulcers, in addition to its anti-inflammatory and analgesic activities [11, 12]. In addition, MTT assay performed with partially purified fractions of* L. ferrea* has shown an inhibitory effect in normal cell growth [8].

Tissue repair and fibrosis may be influenced by directly modulating the inflammatory response and by manipulating endogenous profibrotic mediators that activate important cells in wound site, such as fibroblasts and macrophages [13]. The balance between proinflammatory and anti-inflammatory mediators and the sequester of reactive oxygen species (ROS) are needed to the restoration of normal tissue architecture. Therefore, therapeutic strategies must be engineered in a way that does not negatively affect proregenerative pathways [14].

Some* L. ferrea* compounds are known to be responsible for biological activity, such as phenolic compounds and saponins [15]. Given the popular use of* L. ferrea* and taking into account the need for further studies to investigate its pharmacological properties, this study performed the phytochemical characterization of its fruits' crude extract and fractions and evaluated its anti-inflammatory and antinociceptive activities in an* in vivo *experimental model and its influence on cell viability* in vitro*.

## 2. Material and Methods

### 2.1. Herbal Material

The herbal material was constituted of fruits from* Libidibia ferrea *(Mart. ex Tul.) L.P.Queiroz. var.* ferrea* collected in Limoeiro (PE), Brazil. A voucher specimem was deposited at the Agronomic Institute of Pernambuco (IPA) identified by the number 88145. The material was stabilized by drying in a circulating air oven (40°C) for 7 days before miling and extraction.

### 2.2. Obtaining CE and Enriched Fractions of* Libidibia ferrea *Fruits

The extracts were obtained by turbidysis in the proportion 10% (m/v) using the following as solvent: water (CE) or hydroalcoholic-20-80% ethanol (CE20, CE40, CE60, and CE80, v/v). The extracts were then concentrated in a rotary evaporator to remove the ethanol and frozen at -80°C for three days. The extracts were lyophilized to obtain the aqueous crude extract (CE) and hydroalcoholic extracts (CE20, CE40, CE60, and CE80%, v/v).

Approximately 10.0 g of each CE was reconstituted in water at a ratio of 1:10 (w/v) and then partitioned with 100 ml of ethyl acetate (12 times). Lastly, the fractions were pooled and concentrated to remove the organic solvent, frozen and then lyophilized. The procedure resulted in the following fractions: ethyl acetate (EAF) and aqueous (AqF).

### 2.3. Chromatographic Analysis by High-Performance Liquid Chromatography with Diode Array Detection (HPLC-DAD)

#### 2.3.1. Samples Solutions for the Liquid Chromatography-Analysis

About 50.0 mg of each sample (CE or fractions) previously weighed was transferred to 25.0 ml volumetric flasks. After addition of 20.0 ml of ultrapure water (Elga®), the flasks were then transferred to an ultrasonic bath (Ultracleaner, Unique®) until total dissolution. The volume was made up to 25.0 mL and each sample (CE; hydroalcoholic extracts 20-80% EtOH; or fractions) was diluted to 1 mg/ml with ultrapure water. Gallic acid (96% purity, Sigma®) [16] and ellagic acid from tree bark (95% purity, Sigma®) [16, 17] were used as reference standards. The CE, hydroalcoholic mixture of 20-80% ethanol (CE20, CE40, CE60, and CE80, v/v), fractions, EAF, AqF, and standards were filtered through a polyvinylidene difluoride (PVDF) 0.45 *μ*m membrane (Macherey-Nagel®) prior to liquid chromatography-analysis. The HPLC-DAD analyses were performed in triplicate.

#### 2.3.2. Chromatographic Conditions

The chromatographic analysis followed the previously described method [18]. It was carried out by a Thermo Scientific LC-apparatus (Mod. Ultimate 3000) equipped with a DAD, binary pump (Mod. HPG-3x00RS), degasser, and an autosampler equipped with a 20 *μ*L loop (Mod. ACC-3000). Chromeleon 6.8 software (Dionex®) was used for data acquisition and data processing.

A C_18_-column (250 mm x 4.6 mm inner diameter, 5 *μ*m; Dionex®) protected by a C_18_-guard column was used for the chromatographic separation. A gradient elution was achieved by varying the proportion of solvent B (methanol with 0.05%, v/v, trifluoracetic acid) to solvent A (water with 0.05%, v/v, trifluoracetic acid) at a flow rate of 0.8 ml/min, according to the following gradient program: 10–25% B (10 min), 25–40% B (5 min), 40–70% B (10 min), 75% B (5 min), and 75–10% B (1 min). The analysis was carried out under 23 ± 2°C unsig the wavelengths of 254 nm and 270 nm for detecting of ellagic acid and gallic acid, respectively.

### 2.4. In Vivo Studies

All in vivo tests were approved by the Committee on Ethics in Animal Use/CEUA/Federal University of Rio Grande do Norte/UFRN, protocol number: 001/2015 of the Federal University of Rio Grande do Norte, Brazil. Research protocols and animal care followed the recommendations of the Guide to the Care and Use of Laboratory Animals (NIH Publication No. 85-23, revised 1985).

#### 2.4.1. Mice

Experiments were performed on male Swiss mice 60 days old (40 ± 2.0 g), obtained from the UFRN Vivarium Center of Biosciences and maintained under standard conditions (e.g., 12 h light/dark cycle, 22 ± 0.1°C, and 50–55% humidity) with feed and water appropriate for the species provided* ad libitum*. After being acclimated, the animals fasted for 12 hours with water* ad libitum* prior to the experiments. At the end of the experiment, the animals were euthanized with an overdose of thiopental injected intraperitoneally (100 mg/kg, 0.5%, Tiopentax, Cristália, São Paulo, Brazil).

#### 2.4.2. The Carrageenan-Induced Peritonitis Model

Carrageenan-induced peritoniael inflammation was performed as previously described [19]. Mice were randomized into nine groups (n = 5/group): orally pretreated with a vehicle (0.9% saline solution)/carrageenan (C) group, diclofenac at a dose of 10 mg/kg (D), CE, CE20, CE40, CE60, CE80, EAF, and AqF at the doses of 50 mg/kg, 100 mg/kg, or 200 mg/kg. Thirty minutes later, the mice received 0.25 ml of 1% carrageenan solution (Sigma Aldrich, São Paulo, Brazil) by intraperitoneal (i.p.) injection. A vehicle (1 ml water/10 g, p.o) and a 0.9% sterile saline solution were intraperitoneally injected in the saline (S) group (0.1 ml/10 g) [19]. Four hours later, the mice were intraperitoneally anesthetized with thiopental. Peritoneal exsudate was collected by peritoneal lavage with 3 ml saline solution and used for cell counting in the Neubauer chamber. The samples were then centrifuged at 10,000 for 10 min at 4°C and the supernatant was stored at -80°C for analyses of myeloperoxidase activity (MPO) and for evaluation of malondialdehyde (MDA) and total glutathione levels.

#### 2.4.3. Determination of Myeloperoxidase Activity

MPO activity was measured according Krawisz et al. [20]. An aliquot (100 *μ*L) of each sample was diluted in hexadecyltrimethylammonium bromide buffer (HTAB, Sigma Aldrich, São Paulo, Brazil) and homogenized. The duplicate samples were sonicated for 5 min and then centrifuged at 10,000 rpm for 15 min at 4°C and freeze-thaw three times. To the samples, 200 *μ*l of the staining reagent (o-dianisidine dihydrochloride) was added and the absorbance values at 450 nm were recorded by Spectrocopical UV/VIS analysis (Biotek, São Paulo, Brazil). The absorbance data were interpolated from a standard curve of human neutrophil myeloperoxidase and horseradish peroxidase. One unit of MPO (U) was defined as the activity that degrades 1 nmol/min of hydrogen peroxide at 25°C. The results were expressed as U/*μ*L.

#### 2.4.4. Determination of Total Glutathione Content

Total glutathione content was quantified using the method described by Anderson [21], in which the inflammatory lavage was diluted in a 5% trichloroacetic acid/distilled water solution, following homogenization and centrifugation at 10,000 rpm for 15 min at 4°C. Additionally, 20 *μ*l/well of dithiobisnitrobenzoic acid (DTNB) solution and 140 *μ*l/well ofNADPH were added. The samples were incubated at 30°C for 5 min, and an enzymatic solution of GSHred (Sigma Aldrish, São Paulo, Brazil) was added. The absorbance was evaluated at a wavelength of 412 nm by an UV/VIS Spectrocope (Biotek, São Paulo, Brazil), and total glutathione content of each sample was determined by interpolation of their absorbance on a purified glutathione standard curve (*γ*-L-glutamyl-L-cysteinyl-glycine, Sigma Aldrish, São Paulo, Brazil, G4251). The results were presented in nmol/*μ*l.

#### 2.4.5. Determination of Malondialdehyde Content

MDA production was measured following the method previously described by Esterbauer and Cheeseman [22] to assess lipid peroxidation. The peritoneal fluid samples were diluted in Tris HCl buffer (TRIZMA HCl, Sigma Aldrich, São Paulo, Brazil) in distilled water (20 mM, pH 7.4), following homogenization and centrifugation at 10,000 rpm for 10 min at 4°C. To each sample, chromogenic reagent (10.3 mM 1-methyl-2-phenylindole in 3:1 acetonitrile) and HCl (37%) were added. Subsequently, they were incubated for 40 min at 45°C and centrifuged at 10,000 rpm for 5 min at 4°C. Absorbance was recorded at 586 nm using Spectrocopical UV/VIS analysis (Biotek, São Paulo, Brazil) and interpolated in a standard curve with 1,1,3,3-tetraethoxypropane. The results are shown in nmol/*μ*l.

#### 2.4.6. Evaluation of Antinociceptive Activity

*Hot Plate Testing (Central Analgesic Activity). *The heat sensitivity test was performed in the mice using the Insight hot plate equipment (São Paulo, Brazil) at a temperature of 55 ± 0.5°C (Kuraishi et al., 1983) and the mice that showed a response nociceptive period from 5 to 30 s. The latency time to lick the hind paw or jump was considered as the index of the nociceptive threshold and the cut-off time was 60 s. The preselected mice were randomly divided into groups (n = 5/group): saline 0.9% (10 ml/kg) (negative control group), morphine (10 mg/kg, i.p.), and CE, CE20, CE40, CE60, and CE80 AqF and EAF, the extract, and crude fraction groups received oral doses of 50 mg/kg, 100mg/kg, and 200 mg/kg. Each mouse was then placed on the hot plate and the time taken by the animals to jump or lick one of their hind paws was recorded. Response latencies were recorded at intervals of 30 min, 60 min, 90 min, and 120 min after administration of the respective treatment.

*Acetic Acid-Induced Abdominal Writhing Test (Peripheral Analgesic Activity). *Peripheral antinociceptive activity was evaluated using the acetic acid-induced contortion method [23]. After the 12-hour fast, the male mice were randomly divided into groups (n = 5/group): saline group (10 ml/kg oral saline), oral indomethacin treated group (10 mg/kg), and AA control. The other groups were treated with oral doses of CE, CE20, CE40, CE60, CE80, AqF, and EAF (50 mg/kg, 100 mg/kg, and 200 mg/kg). Thirty minutes after treatment administration, each group was subjected to a nociceptive stimulation with 0.6% acetic acid (v/v, i.p.) to induce the contortions. After 3 min of acetic acid administration, nociceptive responses were verified by counting the number of writhes observed in each mouse for 20 minutes.

#### 2.4.7. Experimental Outcomes

The behavior and deaths of animals were recorded during the experiments.

### 2.5. In Vitro Study

#### 2.5.1. Cell Viability Assay

Mouse embryonic fibroblast 3T3 cell line was obtained from the Culture Collection of the Federal University of Rio de Janeiro and cultured in modified Dulbecco's culture medium (DMEM) supplied with 10% fetal bovine serum (FBS) and 1% antibiotics (penicillin/streptomycin) at 37°C in a 5% CO_2_ atmosphere. An inverted light microscope (NIKON CFI60-Spectrum Bioengineering Medical Hospital LTDA, BR) was used to monitor the cell growth, and cells' maintenance was performed every 3 days. Cell line proliferation was determined by means of a colorimetric assay based on the MTT tetrazolium salt (3-(4,5-dimethylthiazol-2-yl)-2,5-diphenyltetrazolium bromide), which evaluates cell viability by the enzymatic activity of mitochondrial dehydrogenases. 3T3 cells were seeded into 96-well plates at a density of 5.000 cells/cm^2^ followed by incubation for 24 h. Then,* L. ferrea* (AAq, FAq, 80T, 60T, 40T, and 20T) crude extracts and fractions were applied at the concentrations of 0 *μ*g/ml, 10 *μ*g/ml, 15 *μ*g/ml, 20 *μ*g/ml, 25 *μ*g/ml, and 30 *μ*g/ml. Cisplatin (50 *μ*M/ml) was used as a control for cell death and vitamin C as an antioxidant cell control (50 *μ*M/ml). After the treatment period (24h, 48h, and 72h), 100 *μ*l of MTT solution (5 mg/ml) was added to each well. After incubating the cells for 4 h, the medium was removed and 100 *μ*l of ethanol/well was added. The plates were shaken for 15 min and the absorbance was measured in a microplate reader (Epoch-BioTek Instruments Inc., USA) at a wavelength of 570 nm using the Gen5 Data Analysis version 2.0 software (BioTek Instruments Inc., USA).

### 2.6. Statistical Analysis

The normality of the data was verified by using Kolmogorov-Smirnov test. After confirmation of data normality, we applied parametric tests. One-way analysis of variance (ANOVA) was performed to compare means, followed by Bonferroni's post hoc test. The level of statistical significance was set at* p*<0.05. Statistical analysis and graph construction were performed using Graphpad Prism version 5.04.

## 3. Results

### 3.1. Chromatographic Analyses by HPLC-DAD

**Figure 1:**
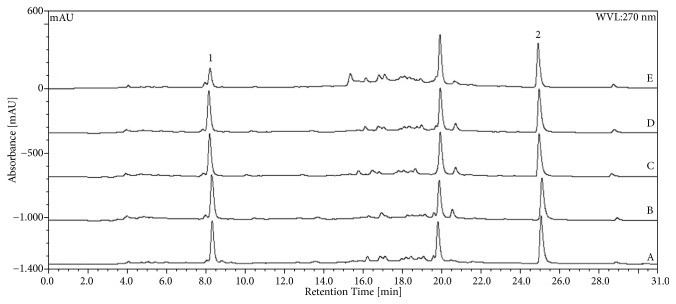
Chromatograms obtained by HPLC for* L. ferrea* crude extracts. Aqueous extract (CE) (A) and hydroalcoholic (v/v) extracts: 20%-CE20 (B), 40%-CE40 (C), 60%-CE60 (D), and 80%-CE80 (E). The markers are indicated by the numbers: (1) gallic acid and (2) ellagic acid.

**Figure 2:**
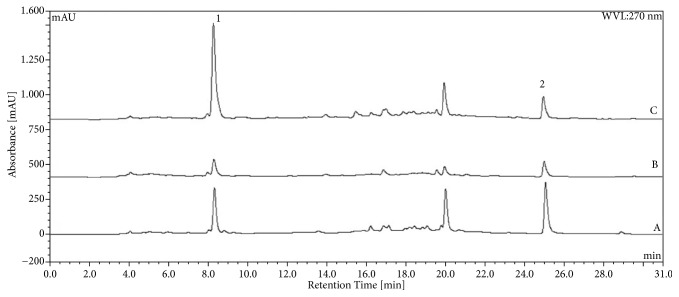
Chromatograms obtained by HPLC-DAD for* L. ferrea* crude aqueous extract (CE) (A); aqueous fraction (AqF) (B); and the ethyl acetate fraction (EAF) (C). The markers are indicated by the numbers: (1) gallic acid and (2) ellagic acid.

The chromatographic analysis showed peaks related to gallic and ellagic acids with retention times of 8.2 and 24.8 min, respectively. Chromatographic profiles for CE, CE20, CE40, CE60, and CE80 of* L. ferrea* are shown in Figure 1. Considering the partitioning of the CE with ethyl acetate, the resulting chromatograms for the EAF and AqF analysis are presented in Figure 2. The calculated values for each of the markers in the crude extracts, the EAF and AqF, are summarized in Table 1. Higher ellagic acid content was observed for EAF (3.06), followed by CE (2.96) and CE40 (2.89). The highest content for gallic acid was found in EAF (12.03), followed by CE20 (4.43) and CE (3.99) (Table 1).

### 3.2. In Vivo Activities

All mice presented a healthy state and were included in the experiments. A total of 100% of the experimental model randomized animals were included in the peritonitis model induced by carrageenan, hot plate testing and acetic acid-induced writhing test. No animals were ruled out from the study. No adverse effects were observed.

#### 3.2.1. Leukocyte Migration Assay

**Figure 3:**
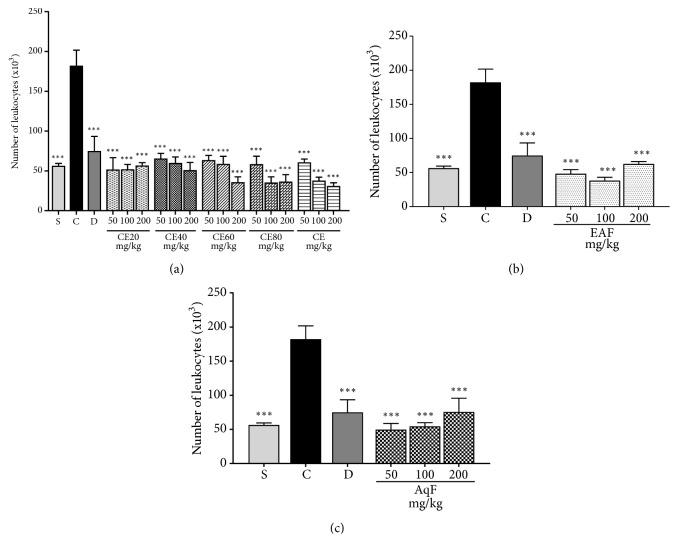
Effects of the crude extract and hydroalcoholic fractions of 20-80% ethanol (a), ethyl acetate fraction (b), and aqueous fraction (c) of* L. ferrea* fruits at different doses on leukocyte migration in the carrageenan-induced peritonitis model. The number of leukocytes^. ^is expressed as mean ± standard error of mean (n = 5). One-way ANOVA test was used to calculate statistical significance, *∗∗∗*p<0.001 versus positive control group. C (carrageenan), S (saline), D (diclofenac), CE20 (20% ethanolic crude extract), CE40 (40% ethanolic crude extract), CE60 (60% ethanolic crude extract), CE (aqueous crude extract), EAF (ethyl acetate fraction), and AqF (aqueous fraction).

Pretreatment with CE, CE20, CE40, CE60, CE80, EAF, and AqF at all doses significantly decreased the number of cells migrating to the inflammation site compared to the positive control group (p<0.001). The groups treated with the extracts and the diclofenac-treated group showed no significant difference in comparison with the negative control group (Figures 3(a), 3(b), and 3(c)).

#### 3.2.2. MPO Activity

**Figure 4:**
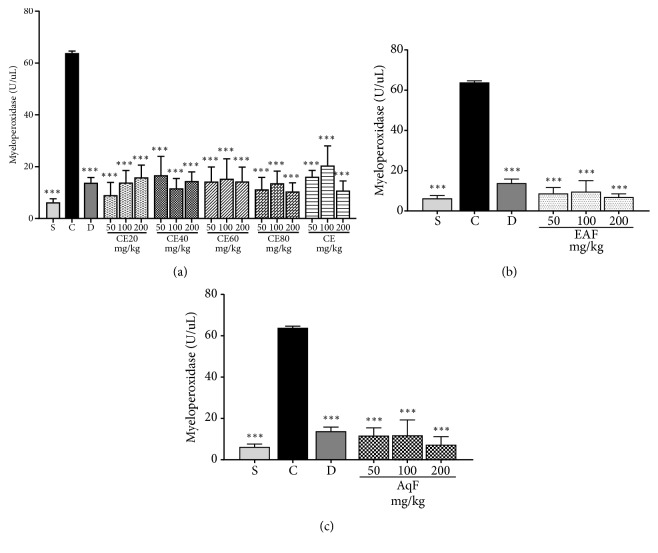
Effects of the crude extract and hydroalcoholic fractions of 20-80% ethanol (a), ethyl acetate fraction (b), and aqueous fraction (c) of* L. ferrea* fruits on myeloperoxidase activity in the carrageenan-induced peritonitis model in mice. Results are expressed as mean ± standard error of mean (n = 5). One-way ANOVA test was used to calculate statistical significance, *∗∗∗*p<0.001 versus positive control group. C (carrageenan), S (saline), D (diclofenac), CE20 (20% ethanolic crude extract), CE40 (40% ethanolic crude extract), CE60 (60% ethanolic crude extract), CE (aqueous crude extract), EAF (ethyl acetate fraction), and AqF (aqueous fraction).

The i.p. injection of carrageenan resulted in the increased MPO activity observed in the positive control group. Treatment with CE and all fractions at all doses was able to cause a decrease in MPO activity when compared to the positive control (p<0.001; Figures 4(a), 4(b), and 4(c)), corroborating the leukocyte migration data.

#### 3.2.3. Total Glutathione Content

**Figure 5:**
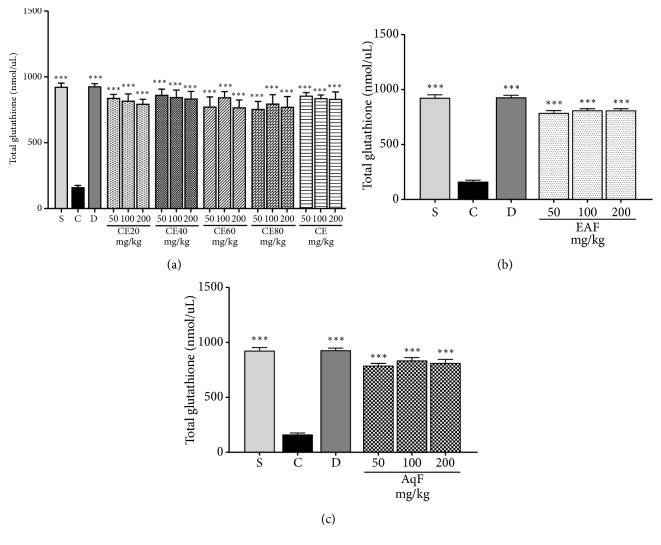
Effects of the crude extract and hydroalcoholic fractions of 20-80% ethanol (a), ethyl acetate fraction (b), and aqueous fraction (c) of* L. ferrea* fruits on total glutathione levels activity in the carrageenan-induced peritonitis model in mice. Results expressed as mean ± standard error of mean (n = 5). ANOVA test used to calculate statistical significance, *∗∗∗*p<0.001 versus positive control group. C (carrageenan), S (saline), D (diclofenac), CE20 (20% ethanolic crude extract), CE40 (40% ethanolic crude extract), CE60 (60% ethanolic crude extract), CE (aqueous crude extract), EAF (ethyl acetate fraction), and AqF (aqueous fraction).

The acute inflammatory process led to a significant decrease in total glutathione levels in the positive control compared to the negative control group. At the tested doses, CE and fractions of* L. ferrea *fruit exerted a beneficial effect observed by the increase of the total* glutathione* levels. As expected, an elevation of glutathione levels was also observed in the group treated with diclofenac (p<0.001). Such results point to an important antioxidant activity exerted by* L. ferrea* (Figures 5(a), 5(b), and 5(c)).

#### 3.2.4. MDA Content

**Figure 6:**
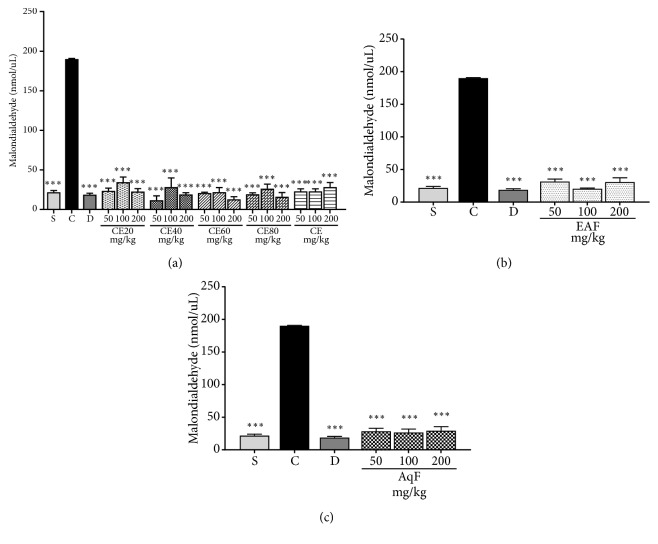
Effects of the crude extract and hydroalcoholic fractions of 20-80% ethanol (a), ethyl acetate fraction (b), and aqueous fraction (c) of* L. ferrea* fruits on malondialdehyde levels in the carrageenan-induced peritonitis model in mice. Results expressed as mean ± standard error of mean (n = 5). One-way ANOVA test used to calculate statistical significance, *∗∗∗*p<0.001 versus positive control group. C (carrageenan), S (saline), D (diclofenac), CE20 (20% ethanolic crude extract), CE40 (40% ethanolic crude extract), CE60 (60% ethanolic crude extract), CE (aqueous crude extract), EAF (ethyl acetate fraction), and AqF (aqueous fraction).

Data revealed that the CE and fractions of* L. ferrea* fruit were able to significantly reduce the MDA levels at all tested doses (Figures 6(a), 6(b), and 6(c)). The positive control group exhibited a significant higher MDA level compared to the treated groups (diclofenac, CE, and fractions) and negative control group (p<0.001), which confirms a reduced lipid peroxidation.

#### 3.2.5. Evaluation of Antinociceptive Activity

*Hot Plate Testing. *Time zero data showed that there was no significant difference between the groups at the beginning of the experiment. Evidence of central analgesic activity was observed in the group treated with morphine at all evaluated times (p<0.05). The group treated with CE presented analgesic activity at a dose of 100 mg/kg at the 90-min time (p<0.05) and at a dose of 200 mg/kg at 60- and 90-min times (p<0.05). The group treated with AqF at the dose of 200mg/kg presented central analgesic action at the 90-minute interval (p<0.05). No continuity between doses and maintenance in the central analgesic activity was observed. There was no significance in the central antinociceptive activity evaluated in the hot plate test at the other time intervals (p>0.05). The other groups did not show relevant central analgesic activity (Table 2).

**Figure 7:**
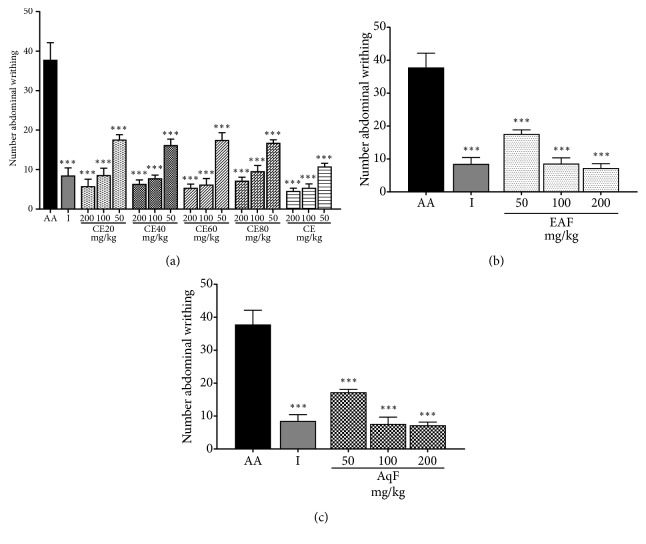
Effects of the crude extract and hydroalcoholic fractions of 20-80% ethanol (a), ethyl acetate fraction (b) and aqueous fraction (c) of* L. ferrea* fruits on nociception in the abdominal writhing model. Results expressed as mean ± standard error of mean (n = 5). One-way ANOVA test used to calculate statistical significance, *∗∗∗*p<0.001 versus AA group. The use of # represents the groups treated with extracts that presented significant difference between them, *∗*p <0.05. AA (acetic acid); I (indomethacin), CE40 (40% ethanolic crude extract), CE60 (60% ethanolic crude extract), CE80 (80% ethanolic crude extract), CE (aqueous crude extract), EAF (ethyl acetate fraction), and AqF (aqueous fraction).

*Abdominal Contractions Induced by Acetic Acid. *The i.p. administration of acetic acid caused a significantly greater number of abdominal writhings in the positive control group compared to the group treated with the extracts at different doses (p<0.001) (Figures 7(a), 7(b), and 7(c)). The number of abdominal writhings is a widely used parameter to evaluate peripheral analgesic activity of a compound. Hence, these results indicate that the CE and fractions of* L. ferrea* present peripheral analgesic activity.

#### 3.2.6. Experimental Outcomes

The records showed no change in the animals' behavior or death during the experiment models.

### 3.3. In Vitro Activity

#### 3.3.1. Cell Viability Assay

**Figure 8:**
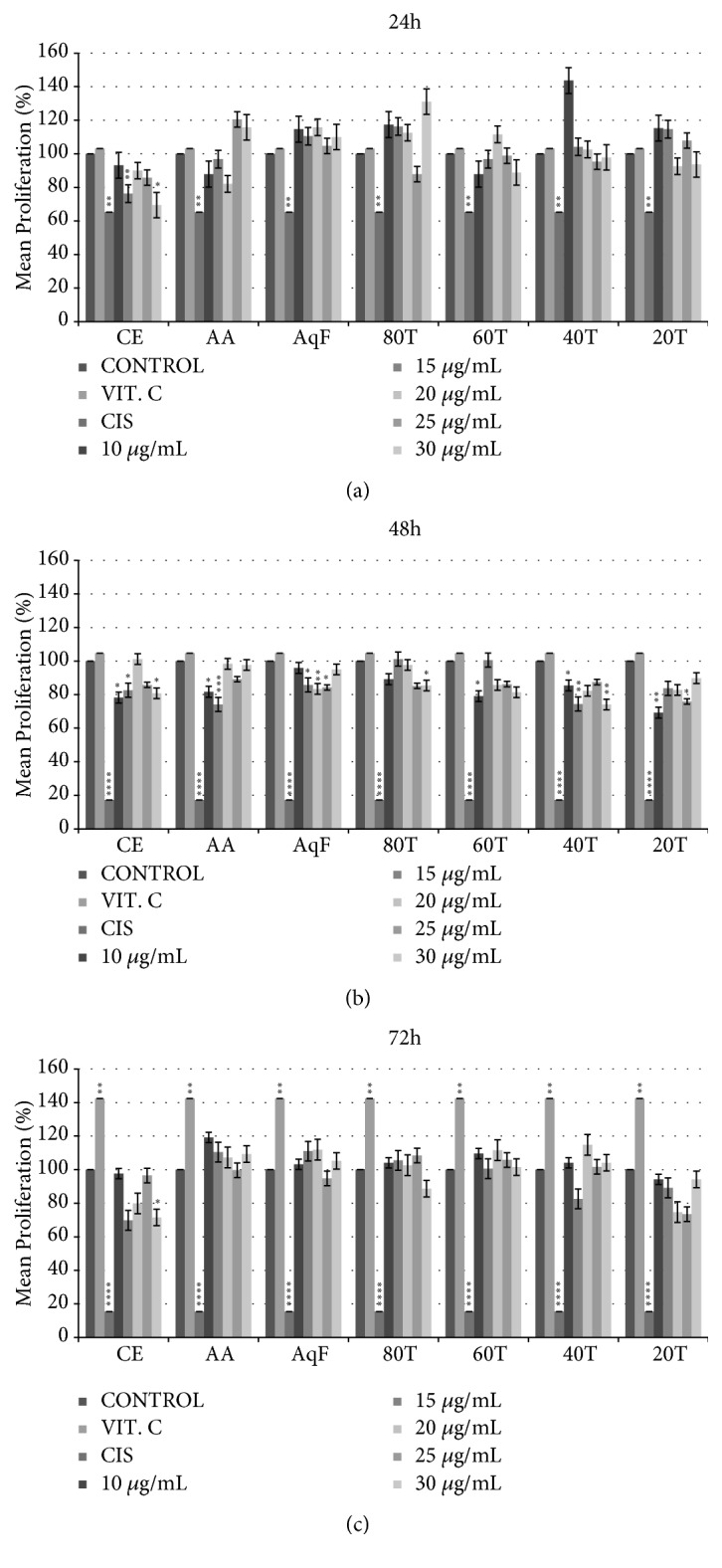
Evaluation of the effect of* Libidibia ferrea* extracts on the 3T3 cell line viability. ((a), (b), and (c)) 24h, 48h, and 72h treatment time (*∗*p<0.05, *∗∗*p<0.01, *∗∗∗*p<0.001, and *∗∗∗∗*p<0.0001 versus control), control (DMEM), VIT C (vitamin C, 50 *μ*M/ml), cis (cisplatine, 50 *μ*M/ml), CE (aqueous crude extract), EAF (ethyl acetate fraction), and AqF (aqueous fraction). CE20 (20% ethanolic crude extract), CE40 (40% ethanolic crude extract), CE60 (60% ethanolic crude extract), and CE80 (80% ethanolic crude extract).

An* in vitro* analysis was performed by MTT colorimetric assay at the 24h, 48h, and 72h times for the 3T3 line to evaluate the effect CE and fractions of of the* L. ferrea *fruits on cell viability (Figure 8). The CE and all fractions were able to cause a significant increase in the cells' viability in the 24-h, 48-h, and 72-h periods compared with the cisplatin- and vitamin C-treated controls (p<0.05).

## 4. Discussion

This study aimed to establish the scientific basis for the popular use of different preparations of* L. ferrea* Linn fruit. In folk medicine, it has been used in the treatment of bronchopulmonary disorders, gastrointestinal disorders, diabetes, rheumatism, inflammation, and wounds in general [7]. The results of the present study indicate that crude and hydroalcoholic extracts of* L. ferrea* Linn fruit have antioxidant, anti-inflammatory, and peripheral antinociceptive effects in an animal experimental model. In addition, they did not present* in vitro *cytotoxic activity, as verified by MTT assay on the 3T3 fibroblasts viability.

The literature shows that no cytotoxicity occurred after 72h of treatment with various doses of gallic acid in 3T3 cells [24]. A MTT assay performed on human umbilical vein endothelial cells (HUVECs) revealed that a higher concentration of ellagic acid may cause the decrease in cell numbers due to the inhibition of cell proliferation, but not cell death [25]. The CE and all fractions were able to cause a significant increase in the 3T3 cell line viability in the three evaluated periods compared with the control. Guerra et al. [26] found similar results, in which crude extracts of* L. ferrea* fruits were able to exert a protective effect on nontumor cell line HEK-293, that presented high rates of cell proliferation and viable cells.

The anti-inflammatory and analgesic properties of some* Fabaceae* family members are described in the literature [8, 11, 12, 27, 28]. A growing number of studies have shown that tannins, the main constituents of* L. ferrea*, exert protective activity in normal cells [29–35]. Among them, gallic and ellagic acids are strongly present in* L. ferrea.* The phytochemical analysis identified the presence of gallic acid and ellagic acid in the CE and fractions of* L. ferrea *fruits, which are thought to be the main components responsible for these effects.

These compounds may act on signaling pathways that are deregulated during pain, inflammation, and oxidative stress. In the carrageenan-induced peritonitis model, acute inflammation is regulated by inflammatory mediators released from neutrophils and macrophages, especially serotonin, bradykinin and histamine in the early hours, and prostaglandins later. Mediators released as a result of exacerbated cell migration to the inflammatory site cause vasodilation and hyperalgesia, induction of erythema formation, edema, and increased permeability [36].

Leukocyte recruitment to the inflammation site is a key factor for the development of an inflammatory process. Therefore, we investigated the anti-inflammatory potential of CE and fractions of* L. ferrea *fruit through a carrageenan-induced peritonitis model. In this model, carrageenan, a potent phlogistic agent, induces the leukocytes migration into the peritoneal cavity. This study showed that CE, CE20, CE40, CE60, and CE80, as well as AqF and EAF fractions from* L. ferrea* fruit, reduced the number of inflammatory cells at the inflammation site. The passage of leukocytes to the inflammation sites is a highly regulated process and represents a potential therapeutic target. The results obtained with the different doses of CE and fractions of* L. ferrea* fruit showed similar results to those observed in the group treated with diclofenac (10 mg/kg), a potent anti-inflammatory drug commonly used in current therapeutic choices.

The MPO enzyme present in the azurophil granules of neutrophils acts on the endothelium, promoting the release of proinflammatory mediators and stimulating the expression of adhesion molecules that cause an increase in vascular permeability and neutrophil adhesion [37]. The CE and fractions of* L. ferrea* fruit at the studied doses resulted in a decrease in MPO activity. Thus, the results show that the decrease in the cellular influx after treatment with CE and fractions is accompanied by a marked decrease in MPO activity.

The pronounced cells recruitment generates oxidative stress, which can be analyzed by determining the total glutathione level, a critical antioxidant defense. Ellagic acid may contribute to the antioxidant potential of medicinal plants. The beneficial effect of CE and fractions of* L. ferrea* fruit at all doses reduced oxidative stress by inhibiting the reduction of total glutathione levels. A low level of total glutathione was identified in the positive control group, indicating increased vulnerability to oxidative stress. EAF and AqF demonstrated an increase in total glutathione levels. These results corroborate those found by Karakurt et al. [38], which investigated the possible role of ellagic acid in the antioxidant potential of the medicinal plant* Epilobium hirsutum* in rat livers.

Another biomarker used to evaluate oxidative stress is MDA, a major secondary product of lipid peroxidation, that indicates oxidative damage to cells and tissues. The important antioxidant activity of both extracts and fractions is confirmed by the significant reduction of MDA levels. Based on the analyzed parameters, the results suggest that CE and fractions have suggestive anti-inflammatory and antioxidant activity. EAF and AqF demonstrate linearity, exhibiting similar activity in the three tested doses. Hydroalcoholic fractions showed a greater decrease in MDA levels at doses of 50 and 200 mg/kg, whereas CE presented similar results at doses of 50 and 100 mg/kg, and lower activity at the dose of 200 mg/kg.

Inflammatory processes are commonly associated with pain as a result of released mediators that stimulate peripheral and central nociceptive pathways. Inflammation mediators can stimulate local sensory neurons, contributing to nociception onset. Prostanoids as well as cytotoxins that are released with tissue injury will act in both the development of the inflammatory process and the hypernociceptive signal [39].

In this sense, we have evaluated the antinociceptive activity of CE and fractions of* L. ferrea *fruit through the abdominal writhing model induced by acetic acid (peripheral) and the hot plate test (central). In the abdominal writhing model, CE and all fractions at all doses showed antinociceptive activity. Peripheral antinociceptive activity of extracts may be related to the their supressive effect on proinflammatory mediators, such as prostaglandins and leukotrienes, due to inhibition of enzymes involved in the inflammatory cascade, namely, cyclooxygenase, lipoxygenase, and phospholipase A2. The treated groups presented evident analgesic activity in the abdominal writhing model, demonstrating that the decrease in the inflammatory process is accompanied by an evident decrease in pain at the peripheral level. The analgesic mechanism of abdominal writhing involves different nociceptive mechanisms such as the process or release of arachidonic acid metabolites via cyclooxygenase and prostaglandin biosynthesis, opioid mechanisms, local peritoneal receptors and mediators related to acetylcholine and histamine, and mediators of the sympathetic system [40].

In a study by Sawada et al. [11],* L. ferrea* aqueous seed extract inhibited the first and second phases of the formalin test (10 mg/kg) and hot plate test (10 mg/kg), indicating that* L. ferrea* also acts on central nociception. In the present study with hot plate model, significant central antinociceptive activity was only found in treatment with CE at the dose of 100 mg/kg at the 90 min time with latency of 22.7 ± 2.7 s and at 200 mg/kg at 60- and 90-min time with latency of 22 ± 3.2, and 23 ± 2.7, respectively and with AqF (22.4 ± 2.6) at the dose of 200 mg/kg at time of 90 min. None of the other groups treated with the* L. ferrea* species exhibited relevant analgesic activity in the hot plate test. Carvalho et al. [27] demonstrated a slight increase in latency time (11.7 ± 0.8 s) using a 100 mg/kg dose of the* L. ferrea* fruit aqueous extract in the hot plate test. The first phase of the formalin test estimulates activity in A-delta fibers, with participation of bradykinin and substance P [11, 41]. Taken together, these findings suggest that the active constituents of* L. ferrea* can promote the antinociceptive effect in A-delta fibers.

## 5. Conclusion

This study evidenced that CE and fractions of* L. ferrea* fruit showed important anti-inflammatory, antioxidant, and peripheral antinociceptive effects* in vivo* and an increased cell viability* in vitro*. The biological activity of the analyzed species is probably related to the presence of bioactive compounds such as gallic and ellagic acids identified in the phytochemical study. The findings of the present study contribute to the ethnopharmacological use of* L. ferrea*.

## Figures and Tables

**Table 1:** Gallic acid and ellagic acid levels in crude extracts and fractions* of L. ferrea *fruit determined by HPLC-DAD. Data are expressed as %; m/m.

Samples	Gallic Acid	Ellagic Acid
CE	2.96 (0.45)	3.99 (2.48)
CE 20% (CE20)	2.78 (0.94)	4.43 (0.24)
CE 40% (CE40)	2.89 (0.42)	3.39 (0.65)
CE 60% (CE60)	2.73 (1.76)	1.61 (0.64)
CE 80% (CE80)	2.61 (0.31)	1.84 (0.32)
AqF	1.22 (0.23)	1.98 (0.28)
EAF	3.06 (1.08)	12.03 (0.25)

Data are displayed as the mean (relative standard deviation) of three independent measurements. CE: crude aqueous extract; AqF: aqueous fraction of crude aqueous extract; EAF: ethyl acetate fraction.

**Table 2:** Central antinociceptive effect of *Libidibia ferrea* crude aqueous extract (CE), ethanolic extracts (CE20, CE40, CE60, and CE80), ethyl acetate (EAF), and aqueous (AqF) fractions at doses of 50, 100, and 200 mg/kg in the hot plate test in mice.

Administered treatment	Initial pain latency	Pain latency at the indicated time points after administration			
	0 s	30 min	60 min	90 min	120 min
Control(saline)	13 + 5.0	6.2 + 6.8	4.8 + 3.3	7.8 + 5.1	6.3 + 2.1

Morfine (10 mg/kg)	8 + 2.3	27 + 3.0^*∗*^	23 + 7.8^*∗*^	25 + 6.4^*∗*^	26 + 2.6^*∗*^

CE 50 mg/kg	7.6 + 2.3	13.2 + 3.4	3.6 + 2.7	8.8 + 2.2	12 + 3.3

CE 100 mg/kg	6.6 + 2.1	8.8 + 2.2	8.0 + 2.0	22. 7 + 2.7^*∗*^	10, 4 + 2.9

CE 200 mg/kg	5.3 + 3.2	10.6 + 2.2	22. 0+ 3.2^*∗*^	23,1+ 2.7^*∗*^	6.6 + 4.1

CE20- 50 mg/kg	5.8 + 3.1	13.6 + 4.0	7.6 + 2.55	8.4 + 2.8	12. 2 + 3.2

CE20-100mg/kg	7.0 + 2,0	7.6 + 2.9	8.0 + 2.6	9.0 + 3.0	10 + 2.2

CE20-200mg/kg	8.0 + 3.2	10.8 + 2.8	8.2 + 3.4	10.4 + 2.7	7.4 + 4.1

CE40-50 mg/kg	5.9 + 2.0	8.4 + 3.2	7.6 + 2.9	9.2 + 3.1	11.8 + 3.5

CE40-100mg/kg	6.0 + 3.2	13,2 + 2.6	7.2 + 2.5	3.6 + 3.1	9.6 + 3.0

CE40-200mg/kg	7.6 + 1.9	17.4 + 2.9	17 + 2.8	12.8 + 2.1	16.8 + 2.8

CE60-50 mg/kg	5.9 + 2.3	5.6 +2.8	9.0 + 3.4	8.0 + 3.6	8.2 + 2.3

CE60-100mg/kg	6.2 + 2.2	8.8 + 4.0	9.0 + 3.4	7.2 + 2.4	9.4 + 2.6

CE60-200mg/kg	6.0 + 2.0	5.8 + 2.2	3.4 + 11.8	12.6 + 2.4	17.2 + 2.9

CE80-50 mg/kg	6.5 + 3.2	8.8 + 3.7	7.6 + 2.9	9.4 + 3.3	11. 4 + 2.8

CE80-100mg/kg	7.0 + 3.2	12.6 + 2.8	8.6 + 2.8	13.4 + 3.3	10.2 + 3.2

CE80-200mg/kg	6.0 + 3.2	16,8 + 3.3	7.2 + 2.4	16.2 + 3.0	14.8 + 3.2

EAF50 mg/kg	4.5 + 2.0	7.25 + 2.3	13.4 + 3.0	8.6 + 0.6	12.2 + 3.2

EAF100 mg/kg	4.5 + 2.0	9.0 + 4.0	8.4 + 3.2	9.8 + 2.7	10 + 2.2

EAF200 mg/kg	5.0 + 2.2.	6,0 + 2,7	4.8 + 3.7	10.4 + 2.7	6.2 + 3.9

AqF50 mg/kg	4.8 + 1.9	6.0 + 2.7	11.2 + 2.9	8.8 + 3.1	12.2 + 3.2

AqF100 mg/kg	5.0 + 2.0	8.8 + 3.6	9.0 + 3.4	9.8 + 2.7	10 + 2.2

AqF200 mg/kg	5.4 + 2.2	6.6 + 3.3	3.4 + 1.8	22.4 + 2.6^*∗*^	7.4 + 4.1

## Data Availability

Data is available from the corresponding author upon reasonable request.

## Abbreviations

CE: Crude extract
AqF: Aqueous fraction
C: Carrageenan
CE20: * Hydroalcoholic crude extract* 20% ethanol
CE40: * Hydroalcoholic crude extract* 40% ethanol
CE60: * Hydroalcoholic crude extract* 60% ethanol
CE80: * Hydroalcoholic crude extract* 80% ethanol
D: Diclofenac
EAF: Ethyl acetate fraction
HPLC-DAD: High-performance liquid chromatography with photodiode array detection
I: Indomethacin
DMEM: Modified Dulbecco's culture medium
FBS: Fetal bovine serum
MTT: 3-(4,5-Dimethylthiazol-2-yl)-2,5-diphenyltetrazolium bromide tetrazolium
CEUA: Ethics Committee on Animal Use
UFRN: Federal University of Rio Grande do Norte
NIH: National Institute of Health
i.p.: Intraperitoneal
MPO: Myeloperoxidase
MDA: Malondialdehyde
HTAB: Hexadecyltrimethylammonium bromide buffer
UV: Ultraviolet
VIS: Visible
GSH: *γ*-L-Glutamyl-L-cysteinyl-glycine
SEM: Standard error of mean
ANOVA: One-way analysis of variance
BAPTA-AM: 1,2-Bis(2-aminophenoxy)ethane-N,N,N′,N′-tetraacetic acid tetrakis(acetoxymethyl ester).
